# Efficacy and safety of SARS-CoV-2 vaccination in patients with inflammatory bowel disease on immunosuppressive and biological therapy: Prospective observational study

**DOI:** 10.1371/journal.pone.0273612

**Published:** 2022-09-02

**Authors:** Martin Wasserbauer, Stepan Hlava, Milan Trojanek, Jan Stovicek, Tomas Milota, Jiri Drabek, Petra Koptová, Andrea Cupkova, Dita Pichlerová, Barbora Kucerova, Stepan Coufal, Radan Keil

**Affiliations:** 1 Department of Internal Medicine, 2nd Faculty of Medicine Charles University in Prague and Motol University Hospital, Prague, Czech Republic; 2 1st Department of Infectious Diseases, 2nd Medical Faculty Charles University in Prague and Hospital Na Bulovce, Prague, Czech Republic; 3 Department of Immunology, 2nd Faculty of Medicine, Charles University and Motol University Hospital, Prague, Czech Republic; 4 Department of Pediatric Surgery, 2nd Faculty of Medicine Charles University in Prague and University Hospital Motol, Prague, Czech Republic; 5 Laboratory of Cellular and Molecular Immunology, Institute of Microbiology of the Czech Academy of Sciences, Prague, Czech Republic; Changhua Christian Healthcare System: Changhua Christian Hospital, TAIWAN

## Abstract

**Background and aims:**

SARS-CoV-2 is a worldwide serious health problem and vaccination seems to have a crucial role in managing the COVID-19 pandemic. The aim of this prospective observational study was to monitor the trend of antibodies against SARS-CoV-2 after vaccination with BNT162b2 (COMIRNATY) in patients with inflammatory bowel disease treated by immunosuppressive and/or biological therapy, demonstrate whether any type of this therapy is associated with poorer production of antibodies against COVID-19 and evaluate the safety of vaccination against COVID-19 in these patients.

**Methods:**

Eighty-seven eligible patients from one tertiary gastroenterological center with inflammatory bowel disease (60 with CD, 27 with UC) treated by immunosuppressive and/or biological therapy from the antiTNFα group were indicated to vaccination against SARS-CoV-2. Effectiveness of vaccination was evaluated by the values of antibodies before and 4 weeks after 2^nd^ dose of vaccine. Additional goal was to evaluate adverse events of vaccination.

**Results:**

Before the 2^nd^ dose of vaccine, geometric mean of SARS-CoV-2 IgG antibodies were 40.7 U/ml in the biological therapy group, 34.8 U/ml in the azathioprine group and 44.8 U/ml in the combination therapy group of patients. The geometric means were 676.5.7 U/ml in the biological therapy group, 614.4 U/ml in the azathioprine group and 500.1 U/ml in the combination therapy group of patients four weeks after 2^nd^ dose. Statistically significant differences between these groups were not proved. Several non-severe local and general adverse events were present in our patients with a majority of these events on the day of vaccine administration and the day after, no anaphylactic reactions were present.

**Conclusions:**

Our measurements proved the efficacy and safety of vaccination against SARS-CoV-2 in patients with inflammatory bowel disease treated by immunosuppressive and/or biological therapy. Statistically significant differences between our groups of patients were not proved.

## Introduction

At the end of 2019 a new coronavirus appeared (then called SARS-CoV-2) which rapidly resulted in a pandemic with serious health and also economic consequences. Infection of SARS-CoV-2 is characterized by rapid transmission of the virus between people [1, 2]. A dysregulated immune response followed by a cytokine storm plays a crucial role in the pathogenesis of COVID-19 infection [3]. Especially in high-risk groups of patients, infection may result in acute respiratory distress syndrome, multiorgan failure and death. Mortality of COVID-19 infection is high and especially elderly patients and patients with comorbidities are at increased risk of death [4].

Inflammatory bowel disease (IBD), including ulcerative colitis (UC) and Crohn’s disease (CD), is a chronic inflammatory disorder of the gastrointestinal tract. The management of IBD typically requires lifelong pharmacological therapy, often including during the past decade immunosuppressive or biological therapy, to induce and maintain remission [5, 6]. Immunosuppressive and biological therapy (especially in combination) is associated with higher risk of viral, bacterial, parasitic or fungal opportunistic infection [7, 8]. Each of these immunomodulators can lead to several type of opportunistic infection despite different mechanisms of action. To this date, few data about COVID-19 infection in patients with IBD has been published. Available but limited data suggest that patients with IBD are not at a higher risk of COVID-19 infection than the general population and immunosuppressive or biological therapy is not associated with a higher mortality rate [9–11].

Direct antiviral treatment for COVID-19 infection is not available as of the date of publication, and therapy for COVID-19 infection is predominantly symptomatic. During the COVID-19 pandemic, countries have focused mainly on preventive methods, namely non-specific (patient triage, social distancing, personal hygiene and disinfection and use of personal protective equipment) and specific (vaccination) ones. Vaccines against COVID-19 infection are available in several different platforms [12] and nowadays widely used. Patients with IBD are also indicated for vaccination against COVID-19 infection to reduce the incidence of infection in this population group and to minimize the acute and chronic consequences of this infection.

The aim of this observational prospective study was to monitor the trend of development of antibodies against COVID-19 after vaccination in patients with IBD, who are being treated by immunosuppressive and/or biological therapy from the group of Tumor necrosis factor α inhibitors (antiTNFα), and demonstrate whether any type of chronic immunosuppressive/biological therapy for IBD was associated with poorer production of antibodies against COVID-19. The second goal of the study was to evaluate the safety of vaccination against COVID-19.

## Materials and methods

### Patients

A prospective observational study was performed at the Gastroenterology Department in Motol University Hospital in Prague, Czech Republic in outpatients > 18 years of age. Patients with CD or UC were eligible to participate in the study if they used a medication containing immunosuppressive therapy (azathioprine) and/or biological therapy from the antiTNFα group (infliximab, adalimumab, golimumab) and were indicated to vaccination against SARS-CoV-2. In order to ensure that results were not affected by potentially confounding factors, exclusion criteria were as follows: chronic or recent (≤ 2 weeks before vaccination) use of corticosteroids and infection of COVID-19 before vaccination. During the study, patients received the usual care from their gastroenterologists, which was not modified because of the study.

### Ethical statement

The study protocol was approved by the ethics committee of the Second Faculty of Medicine, Charles University in Prague, Czech Republic. All participants of our study (patients over 18 years of age) signed an informed consent.

### Methods

Eligible patients (men and women) with CD or UC were indicated to vaccination against SARS-CoV-2 with BNT162b2 (COMIRNATY, Pfizer-BioNTech) COVID-19 vaccine according to the indication criteria listed in the Summary of Product Characteristics in the Czech Republic. No patient selection for vaccination or inclusion into our study was done. All patients of our gastroenterological center were gradually vaccinated. All patients treated by immunosuppressive (azathioprine) and/or biological therapy (antiTNFα group), who agreed to participate with our study, were included. Patients received two doses of the vaccine about 3 weeks apart. Patients agreed to the vaccination and confirmed their consent by signing an informed consent.

Information about patients was collected at the beginning of the monitoring in order to collect information on baseline demographics and characteristics. In patients enrolled in the study, serum levels of COVID-19 antibodies in the IgG classes were collected three times during the entire follow-up: at baseline before the 1st dose of vaccine, before the 2nd dose of vaccine (approximately 3 weeks after the 1st dose) and 4 weeks after the 2nd dose of the vaccine. Based on the value of antibodies against SARS-CoV-2 before the 2nd dose of vaccine and 4 weeks after the 2nd dose of the vaccine, patients were divided into 3 groups: non-responders (SARS-CoV-2 antibodies IgG less than 100 U/ml), partial responders (SARS-CoV-2 antibodies IgG 100–499 U/ml) and responders SARS-CoV-2 antibodies IgG 500 U/ml and more).

An immune-enzymatic kit (EIA COVID-19 RBD IgG CoRG96) was used for laboratory analysis to determine IgG antibodies against the receptor-binding domain (RBD) of SARS-CoV-2 virus in human serum. RBD is a key part of SARS-CoV-2 virus. RBD, a subunit of the spike S1 protein, binds specifically to the angiotensin-converting enzyme 2 of the host cell and allows virus to entry into cells, which could lead to infection. This binding is highly correlated with the formation of neutralizing antibodies. This kit allows the detection of specific IgG class antibodies in a serum sample by enzyme immunoassay. The labeled antibody is a peroxidase-conjugated animal immunoglobulin fraction against human IgG. Peroxidase activity is determined using a substrate with tetramethylbenzidine, which turns blue in case of positivity. The whole reaction is stopped with a stop solution. The blue color changes to yellow. The intensity of the yellow color is measured on a photometer (at a wavelength of 450 nm) and is proportional to the concentration of specific IgG antibodies present in the sample. In our measurements, the concentration of IgG against SARS-CoV-2 was measured up to the maximum limit of 1000 U / ml, higher concentrations were no longer measured.

Questionnaires focused on the safety of the administered vaccine (S1 Fig) were anonymously distributed to randomly selected patients from the study group. This questionnaire was created by our group, focused directly on the parameters under our consideration and intention. Questionnaires were completed on the day of vaccine administration and for the following 7 days—after the 1^st^ and also after the 2^nd^ dose of vaccine (a total of 16 days, 8 days after both doses). The questionnaires were designed the same for each day. This questionnaire consisted of 3 parts, which measured three forms of side effects of the SARS-CoV-2 vaccine: local reactions (pain and erythema at the injection site), general symptoms (body temperature, headache, arthralgia, myalgia, fatigue and others) and general well-being (on a scale from 0–100).

The primary outcome of the study was to monitor the development of antibodies against SARS-CoV-2 after vaccination in patients with IBD, who were treated by immunosuppressive and/or biological therapy from the antiTNFα group, and demonstrate whether any type of chronic immunosuppressive/biological therapy for IBD was associated with poorer production of antibodies against SARS-CoV-2. The secondary outcome of the study was to evaluate the safety of vaccination against SARS-CoV-2. This study was designed as prospective observational study.

### Statistical analysis

Stata 14 software (StataCorp. 2015. Stata Statistical Software: Release 14. College Station, TX: StataCorp LP) was used for statistical analysis. A censored linear regression model with random effects was used for data comparing IgG antibody levels and applied to logarithmic IgG data (distribution of measured values is asymmetric). Censored regression can work with data with incomplete information, i.e, with the indication that the observation is above a certain limit (here, for example, IgG > 1000 U/ml). The model also takes into account, through random effects, that these are repeated observations on the same individuals. Fisher’s exact test was used to determine whether there is any difference in proportions between groups.

## Results

### Patient demographics and disposition

Eighty-seven eligible patients (52 men and 35 women) who had been diagnosed with IBD (60 with CD, 27 with UC) were enrolled (Table 1). Between UC and CD, there were no statistically significant differences in sex representation (p = 0.480), age (p = 0.244) or therapy types (p = 0.243). Patients were between 23 and 74 years of age, and the median of age was 50. Patients were divided into three groups according to their therapy: biological therapy from the antiTNFα group only (47 patients– 36 with CD and 11 with UC), immunosuppressive therapy with azathioprine only (28 patients—17 with CD and 11 with UC) and a combination of biological therapy from the antiTNFα group and azathioprine group (12 patients—7 with CD and 5 with UC). In the group of patients with biological therapy, 25 patients were treated with infliximab, 21 patients with adalimumab and 1 patient with golimumab. A total of 35 patients were treated with biological therapy in a classic dosing schedule and 12 patients were treated in an intensified regimen (higher dose or shortened interval). In the group of patients with a combination of biological therapy from the antiTNFα group and azathioprine group, 7 patients were treated with infliximab, 5 patients with adalimumab and no patients with golimumab. In the group of patients who were using azathioprine, the median dosage per day was 1.4 mg/kg in the range of 0.3–2.8 mg/kg. Finally, the median interval between vaccine doses was 23 days, ranging from 21 to 25 days.

**Table 1:** Basic demographics and disposition of patients.

		N (%)	Crohn´s disease N (%)	Ulcerative colitis N (%)
Sex	Men	52 (59.8)	34 (56.7)	18 (66.7)
	Women	35 (40.2)	26 (43.3)	9 (33.3)
IBD therapy	Biological therapy	47 (54.0)	36 (60.0)	11 (40.7)
	Azathioprine	28 (32.2)	17 (28.3)	11 (40.7)
	Biological therapy and azathioprine	12 (13.8)	7 (11.7)	5 (18.5)
Total		87	60	27

### Measure of antibodies against SARS-CoV-2

**Fig 1:**
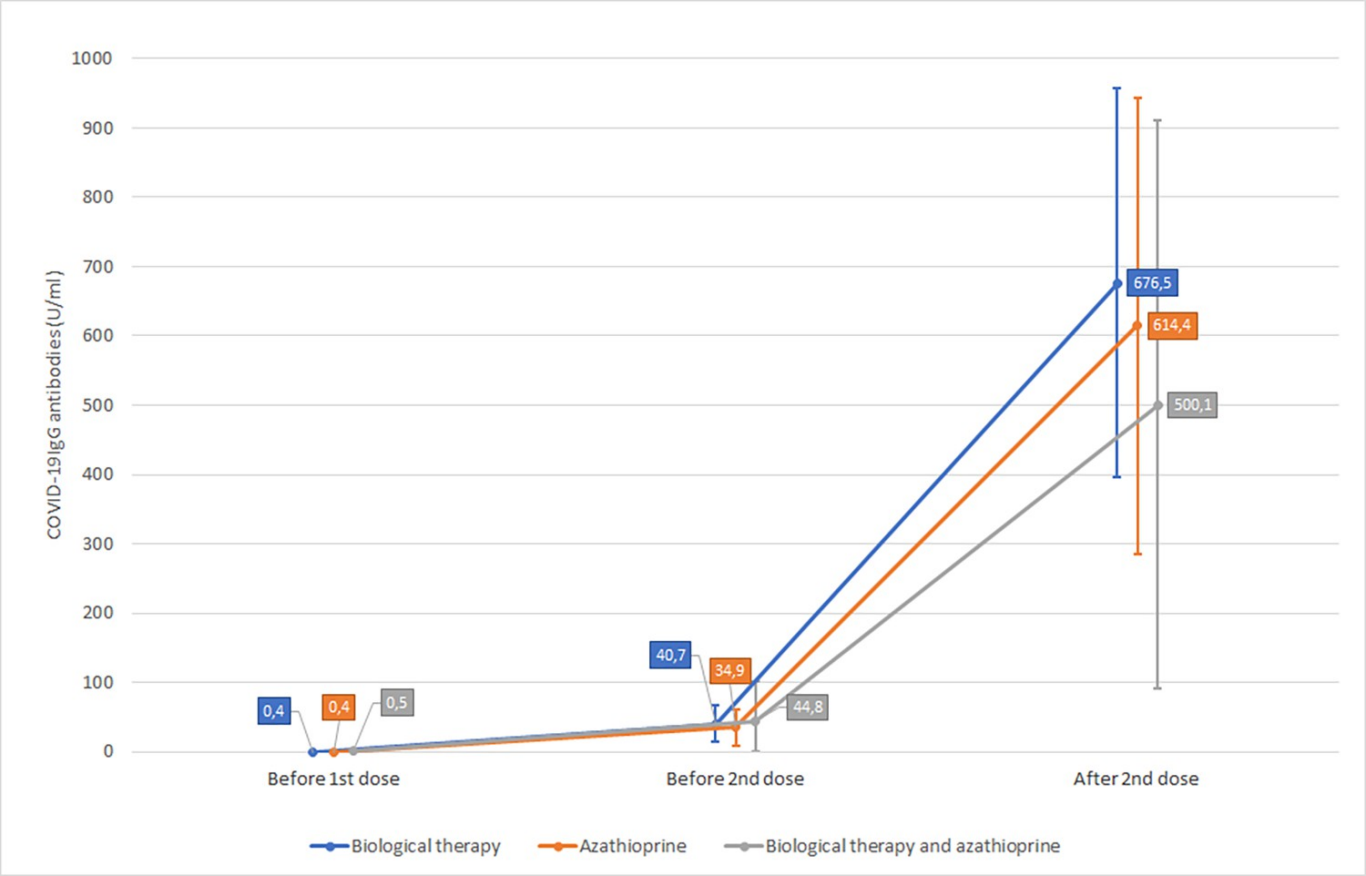
Comparison of IgG antibodies values (geometric means) against SARS-CoV-2 in all 3 groups of patients.

IgG antibodies against SARS-CoV-2 were measured according to our protocol in all 3 groups of patients, geometric means (standard errors) (Fig 1) (Table 2):

1. Group of patients treated with biological therapy (n = 47). All patients had negative values of antibodies IgG against SARS-CoV-2 before the 1^st^ dose of vaccine against SARS-CoV-2—geometric mean of values of antibodies IgG against SARS-CoV-2 was 0.4 U/ml (0.1). Three weeks after 1^st^ dose (before 2nd dose) geometric mean of values of antibodies IgG against SARS-CoV-2 was 40.7 U/ml (13.0). Four weeks after 2^nd^ dose geometric mean was 676.5 U/ml (142.8).
2. Group of patients treated with azathioprine (n = 28). All patients had negative values of antibodies IgG against SARS-CoV-2 before the 1^st^ dose of vaccine against SARS-CoV-2 –geometric mean of values of antibodies IgG against SARS-CoV-2 was 0.4 U/ml (0.1). Median and geometric mean of values of antibodies IgG against SARS-CoV-2 three weeks after 1^st^ dose was 34.9 U/ml (13.0). Four weeks after 2^nd^ dose geometric mean was 614.4 U/ml (168.1).
3. Group of patients treated with the combination of biological therapy and azathioprine (n = 12). All patients had negative values of antibodies IgG against SARS-CoV-2 before the 1^st^ dose of vaccine against SARS-CoV-2 –geometric mean of values of antibodies IgG against SARS-CoV-2 was 0.5 U/ml (0.2). Three weeks after 1^st^ dose geometric mean of values of antibodies IgG against SARS-CoV-2 was 44.8 U/ml (29.0). Four weeks after 2^nd^ dose geometric mean was 500.1 U/ml (209.1).

**Table 2:** Values of IgG antibodies against SARS-CoV-2 in 3 groups of patients, geometric means (standard errors).

IBD therapy	Patients	Before 1st dose, geometric mean	Before 2nd dose, geometric mean	After 2nd dose, geometric mean	After 2nd dose, estimation of geometric mean
Biological therapy	Total	0.4 (0.1)	40.7 (13.0)	676.5 (142.8)	1759.1 (543.5)
	Crohn´s disease	0.3 (0.1)	32.9 (10.1)	610.5 (149.3)	1404.4 (482.8)
	Ulcerative colitis	0.4 (0.2)	166.3 (86.2)	946.4 (365.8)	3603.0 (2361.7)
Azathioprine	Total	0.4 (0.1)	34.9 (13.0)	614.4 (168.1)	2123.1 (897.1)
	Crohn´s disease	0.4 (0.2)	34.0 (15.0)	504.4 (179.5)	1371.8 (702.6)
	Ulcerative colitis	0.4 (0.2)	43.7 (22.2)	833.2 (322.0)	4374.3 (3144.7)
Biological therapy and azathioprine	Total	0.5 (0.2)	44.8 (29.0)	500.1 (209.1)	1492.2 (925.5)
	Crohn´s disease	0.3 (0.2)	44.6 (30.8)	494.5 (274.3)	1160.2 (896.4)
	Ulcerative colitis	0.7 (0.5)	69.1 (53.3)	508.0 (291.2)	1924.1 (1845.9)

Statistically significant increases in the geometric means of values of antibodies IgG against SARS-CoV-2 in all three studied groups of patients (biological therapy only, azathioprine only and combination therapy) were proved before the 1st dose vs. three weeks after 1st dose (before 2nd dose) (p <0.001) and three weeks after 1st dose vs. one month after 2nd dose (p <0.001). The evaluation within IBD diagnoses is described in Table 2.

### Comparison of effectiveness of vaccination against SARS-CoV-2 between monitored groups of patients

Comparison of geometric means in the trend between two time periods and two selected groups:

1. Biological therapy group vs. group of patients with azathioprine—statistically significant differences in antibodies IgG trends were not proved before 1st and 2nd doses (p = 0.470) and nor before 2nd and one month after 2nd dose (p = 0.450).
2. Biological therapy group vs. group of patients with a combination of biological therapy and azathioprine—statistically significant differences in antibodies IgG trends were not proved before 1st and 2nd doses (p = 0.810) and nor before 2nd and one month after 2nd dose (p = 0.710).
3. Combination of biological therapy and azathioprine group vs. group of patients with only azathioprine—statistically significant differences in antibodies IgG trends were not proved before 1st and 2nd doses (p = 0.780) and nor before 2nd and one month after 2nd dose (p = 0.380).

Statistically significant differences in the values of antibodies against SARS-CoV-2 between our groups of patients (biological therapy only, azathioprine only and combination therapy) were not proved.

### Effectiveness of vaccination against SARS-CoV-2

**Fig 2:**
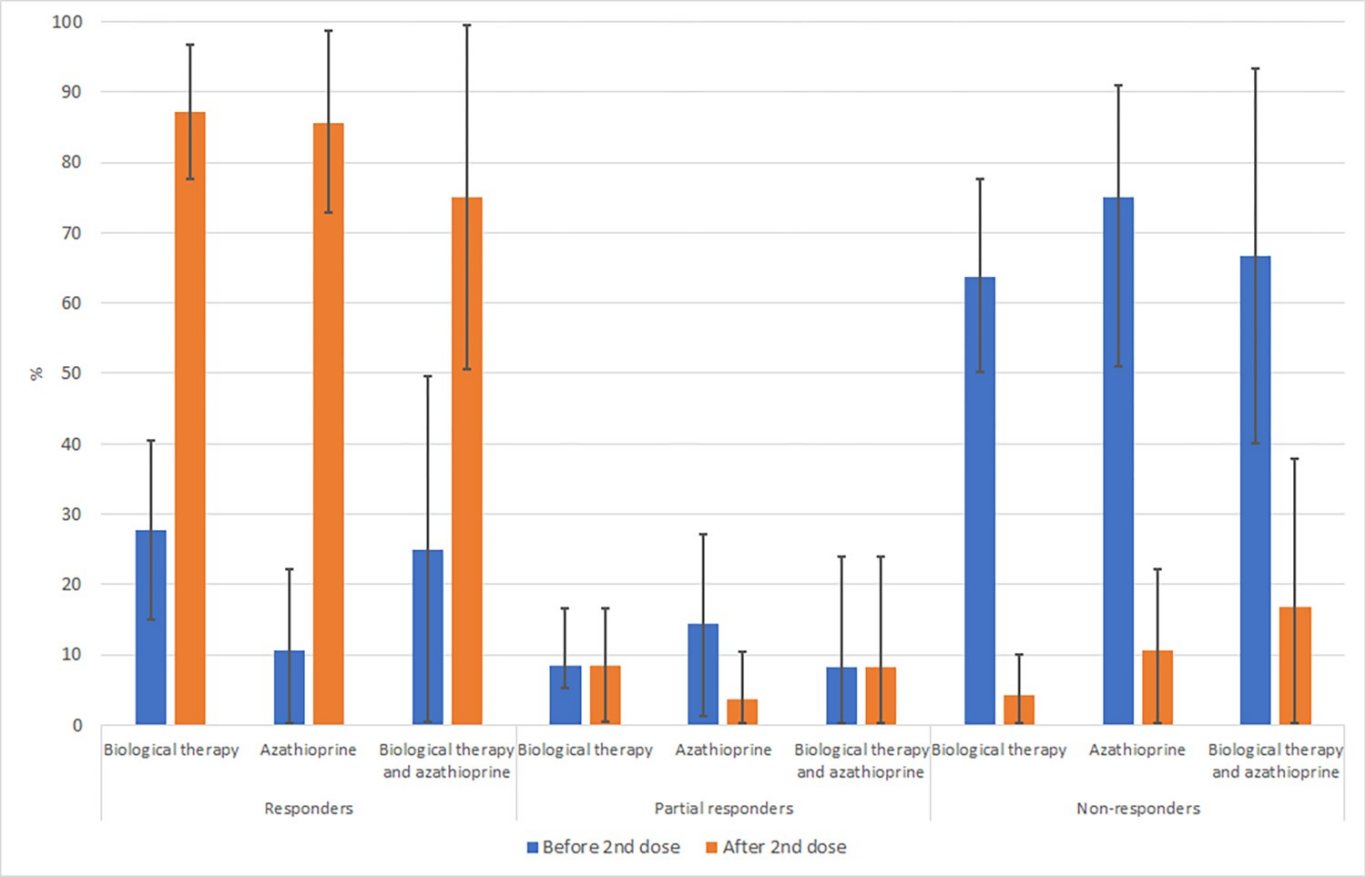
Comparison of responders, partial responders and non-responders between all 3 groups of patients based on the value of antibodies against SARS-CoV-2.

Based on the value of antibodies against SARS-CoV-2, patients were divided into responders, partial responders and non-responders (Fig 2):

1. In the group of patients treated with biological therapy (n = 47, 36 with CD and 11 with UC), only 13 patients (27.7%)(8 with CD and 5 with UC) were responders to vaccination already after the 1st dose, 4 patients (8.5%)(all with CD) were partial responders and 30 patients (63.8%)(24 with CD and 6 with UC) were non-responders. A total of 41 patients (87.2%)(30 with CD and 11 with UC) were responders to vaccination after the 2nd dose, 4 patients (8.5%)(all with CD) were partial responders and 2 patients (4.3%)(all with CD) were non-responders four weeks after the 2nd dose of vaccine against COVID-19.
2. In the group of patients treated with azathioprine (n = 28, 17 with CD and 11 with UC), only 3 patients (10.7%)(2 with CD and 1 with UC) were responders to vaccination already after the 1st dose, 4 patients (14.3%)(2 with CD and 2 with UC) were partial responders and 21 patients (75.0%)(13 with CD and 8 with UC) were non-responders. A total of 24 patients (85.7%)(14 with CD and 10 with UC) were responders to vaccination after the 2nd dose, 1 patient (3.6%)(with UC) was a partial responder and 3 patients (10.7%)(all with CD) were non-responders four weeks after the 2nd dose of vaccine against COVID-19.
3. In the group of patients treated with a combination of biological therapy and azathioprine (n = 12, 7 with CD and 5 with UC), 3 patients (25.0%)(1 with CD and 2 with UC) were responders to vaccination already after the 1st dose, 1 patient (8.3%) (with CD) was a partial responder and 8 patients (66.7%)(5 with CD and 3 with UC) were non-responders. A total of 9 patients (75.0%)(5 with CD and 4 with UC) were responders to vaccination after the 2nd dose, 1 patient (8.3%)(with CD) was a partial responder and 2 patients (16.7%)(1 with CD and 1 with UC) were non-responders four weeks after the 2nd dose of vaccine against COVID-19.

Patients treated only by azathioprine (n = 28) were also divided into 2 groups based on the dose of azathioprine. A total of 6 patients had a dose of azathioprine of 2mg/kg/day and more, of which 1 patient (16.7%) was a non-responder after the 2nd dose of vaccination and 5 patients (83.3%) were responders or partial responders. In the group of 22 patients with azathioprine dose below 2mg/kg/day, 2 patients (9.1%) were non-responders and 20 patients (90.9%) were responders or partial responders 4 weeks after the 2nd dose of vaccination. Statistically significant differences between these two groups of patients were not proved (p = 0.530).

Patients treated only by biological therapy (n = 47) were also divided into 2 groups based on a normal or intensified dosing regimen. A total of 12 patients had an intensified regimen of biological therapy, of which 1 patient (8.3%) was a non-responder after the 2nd dose of vaccination and 11 patients (91.7%) were responders or partial responders. In the group of 35 patients with a classic dosing schedule of biological therapy, 1 patient (2.9%) was a non-responder and 34 patients (97.1%) were responders or partial responders 4 weeks after the 2nd dose of vaccination. Statistically significant differences between these two groups of patients were not proved (p = 0.450).

### Safety and tolerability of COVID-19 vaccine BNT162b2

**Fig 3:**
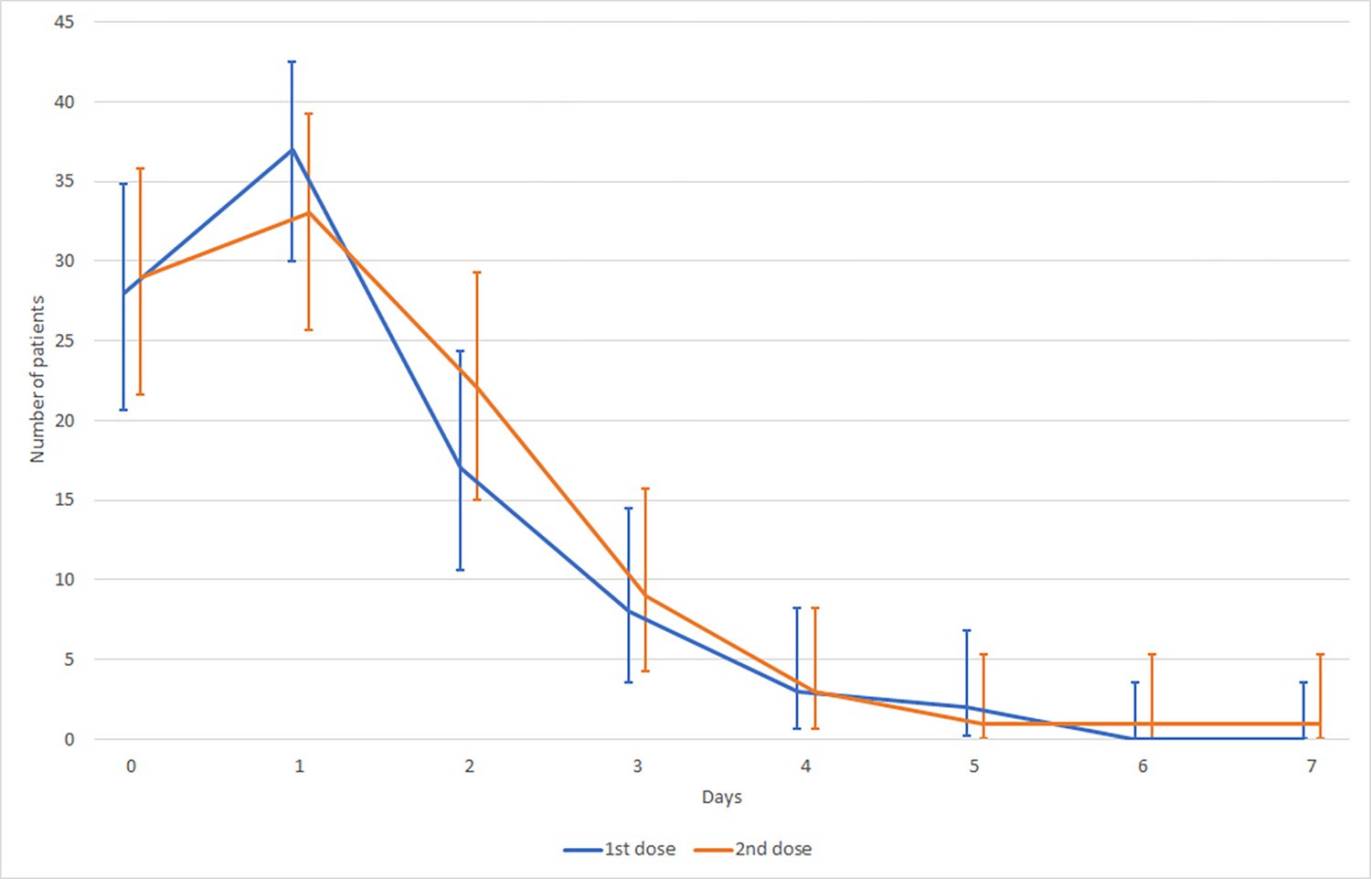
Local adverse reactions after SARS-CoV-2 vaccination—development in time.

**Fig 4:**
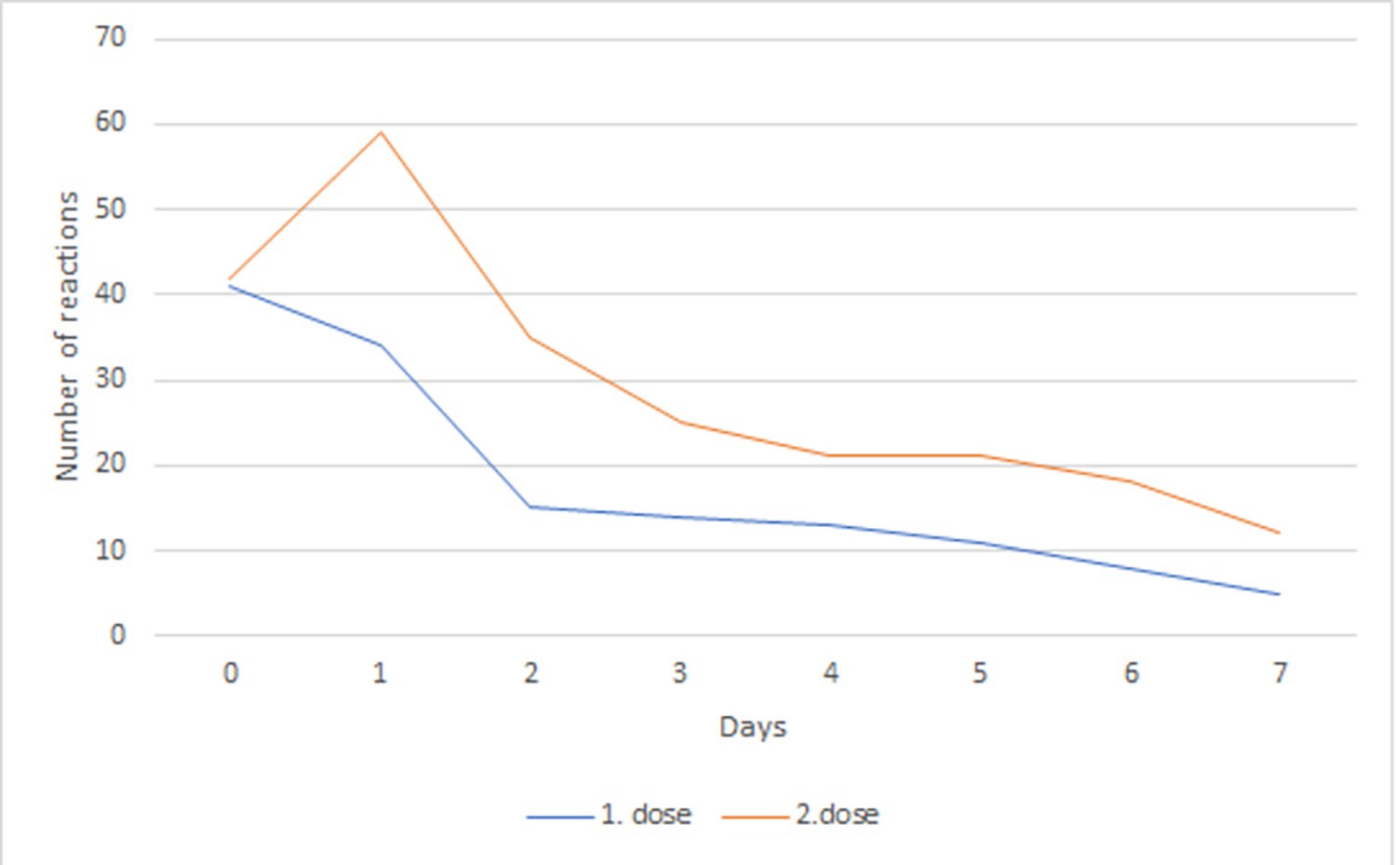
General adverse reactions after SARS-CoV-2 vaccination—development in time.

**Fig 5:**
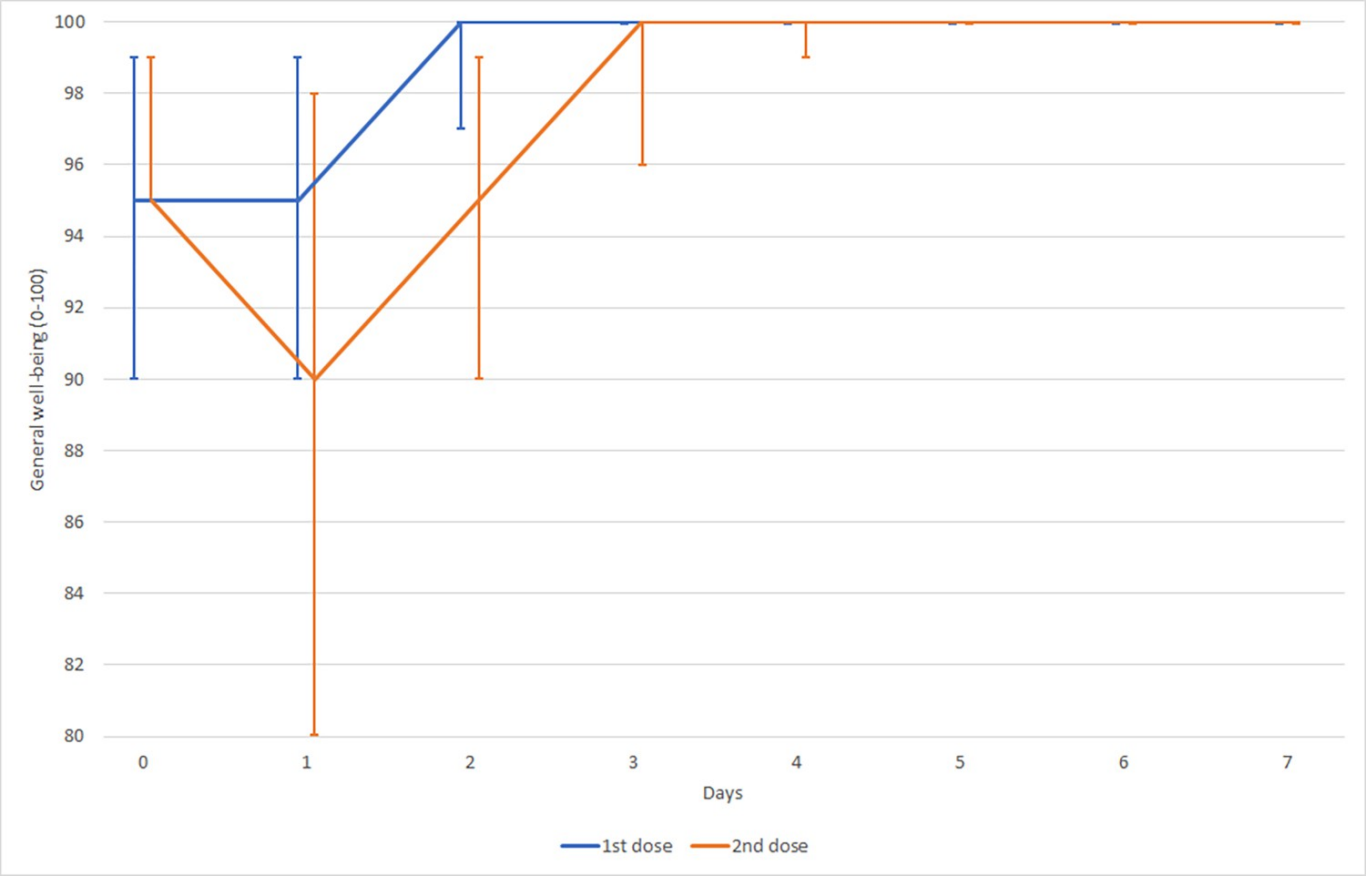
General well-being after SARS-CoV-2 vaccination—development in time.

A total of 49 questionnaires (32 patients with CD and 17 with UC) were evaluated in these three aspects of safety and tolerability of SARS-CoV-2 vaccine BNT162b2:

1. Local reactions (pain and/or erythema at the injection site):
  - Most local reactions were observed on the day of administration (day 0) and the following day (day 1) after the 1^st^ and 2^nd^ dose of vaccine. In the following days, there was a significant decrease in the incidence of local reactions (Fig 3). Erythema was observed on day 0 in 28 patients (57,2%) after the 1^st^ and 2^nd^ dose of vaccine (median of erythema severity was 3 and 2, respectively). On day 1, the progression of erythema frequency in the study group of patients after the 1^st^ and 2^nd^ dose of vaccine was observed in 37 (75,5%) and 32 patients (65,3%), respectively (median severity of erythema was 2 and 3, respectively). Local pain was observed on day 0 after the 1^st^ and 2^nd^ doses of vaccine in 2 patients (4,2%) and 1 patient (2,1%), respectively (median erythema severity was 3 and 2, respectively).
2. General symptoms (body temperature, headache, arthralgia, myalgia and others):
  - A majority of general reactions were observed on the day of administration (day 0) and the following day (day 1) after the 1^st^ and 2^nd^ dose of vaccine with significant decrease following in the days afterward (Fig 4). Mainly fever, myalgia, arthralgia, cephalgia and fatigue were observed during our monitoring after the first and second dose of vaccine (Table 3). Body temperature ≥ 37,0°C in evening was present after the 1^st^ dose and after the 2^nd^ dose of vaccine on day 0 in 5 (10,2%) and 6 (12,2%) patients, respectively; on day 1 in 2 (4,1%) and 7 (14,3%) patients, respectively; on day 7 in 2 (4,1%) and 1 patient (2,0%), respectively. Myalgia was present after the 1^st^ dose and after the 2^nd^ dose of vaccine on day 0 in 7 (14,3%) and 5 (10,2%) patients, respectively; on day 1 in 6 (12,2%) and 13 (26,5%) patients, respectively; on day 7 in 0 (0,0%) and 2 patients (4,1%), respectively. Arthralgia was present after the 1^st^ dose and after the 2^nd^ dose of vaccine on day 0 in 6 (12,2%) and 5 (10,2%) patients, respectively; on day 1 in 5 (10,2%) and 13 (26,5%) patients, respectively; on day 7 in 0 (0,0%) and 2 patients (4,1%), respectively. Cephalgia was present after the 1^st^ dose and after the 2^nd^ dose of vaccine on day 0 in 10 (20,4%) and 11 (22,4%) patients, respectively; on day 1 in 13 (26,5%) and 12 (24,5%) patients, respectively; on day 7 in 0 (0,0%) and 2 patients (4,1%), respectively. Fatigue was present after the 1^st^ dose and after the 2^nd^ dose of vaccine on day 0 in 10 (20,4%) and 12 (24,5%) patients, respectively; on day 1 in 6 (12,2%) and 10 (20,4%) patients, respectively; on day 7 in 1 (2,1%) and 4 patients (8,2%), respectively. The other symptoms were: pruritic rash, nausea, vertigo, back pain, painful lymphadenopathy and dyssomnia. These other symptoms were described only in individual patients and not in more than 2 patients from our observed spectrum (less than 5% of patients). There was no reported severe allergic reaction in terms of anaphylaxis.
3. General well-being (in scale from 0–100):
  - General well-being was monitored after the 1^st^ and 2^nd^ doses of vaccine (0 worst, 100 best). The first dose was better tolerated by patients than the 2^nd^ dose of vaccine (Fig 5).

**Table 3:** General adverse reactions in time.

Days	Number of patients with body temperature ≥ 37,0°C in evening after 1.dose / after 2.dose	Number of patients with myalgia after 1.dose / after 2.dose	Number of patients with artralgia after 1.dose / after 2.dose	Number of patients with cefalgia after 1.dose / after 2.dose	Number of patients with fatigue after 1.dose / after 2.dose	Number of patients with other general symptoms after 1.dose / after 2.dose	Number of patients with the need for emergency farmacotherapy after 1.dose / after 2.dose
0	5 / 6	7 / 5	6 / 5	10 / 11	10 / 12	3 / 3	3 / 5
1	2 / 7	6 / 13	5 / 13	13 / 12	6 / 10	2 / 4	5 / 6
2	0 / 1	2 / 10	3 / 8	5 / 5	4 / 6	1 / 5	2 / 2
3	1 / 0	2 / 5	3 / 4	4 / 6	2 / 5	2 / 5	1 / 0
4	2 / 1	1 /2	3 / 4	4 / 3	1 / 6	2 / 5	2 / 1
5	0 / 1	1 / 4	2 / 4	4 / 4	2 / 4	2 / 4	1 / 2
6	1 / 1	0 / 4	1 / 3	3 / 3	1 / 4	2 / 3	0 / 1
7	2 / 1	0 / 2	0 / 2	0 / 2	1 / 4	2 / 1	0 / 1

## Discussion

A wide spectrum of protective mechanisms has been implemented in the management of prevention of COVID-19 infection, but vaccination is considered to be the most promising tool for managing present pandemic and preventing future outbreaks of this disease [13]. During a relatively short period of time several vaccines became available for use in preventing especially severe forms of COVID-19 infection. Severe form of COVID-19 infection predominantly occurs in high-risk patients (advanced age, medical comorbidities and use of immunomodulatory therapy) [14–18]. Therefore, the issue of vaccination against SARS-CoV-2 in immunocompromised patients or in patients who are using immunomodulatory therapy, such as patients with IBD, is widely recommended with respect to the potential for severe COVID-19 infection. Generally, patients with IBD in remission are not at a higher risk for SARS-CoV-2 infection and these patients should continue their chronic therapy to sustain remission [19]. Risk factors for severe COVID-19 in IBD patients seems to be increasing age, more than 2 comorbidities, use of systemic glucocorticoids and mesalamine/sulfasalazine [11]. Biological therapy with anti-TNF agents is not associated with severe form of COVID-19 [11]. On the other hand, azathioprine, especially in combination with anti-TNF biological therapy, seems to be associated with increased risk of severe COVID-19 [20]. According to these data, the combination therapy of azathioprine with anti-TNF biologic therapy is associated with a worse course of COVID-19 infection than monotherapy with this type of biologic therapy [20].

The immunogenicity of SARS-CoV-2 vaccines appears to be lowered in immunocompromised patients compared to the general population. In studies with transplant recipients, suboptimal immunogenicity with SARS-CoV-2 vaccination was evident [21, 22]. Prevalence of anti–SARS-CoV-2 antibodies at 4 weeks after the 2nd dose of vaccine in all transplant patients was present only in 34% of patients [21]. Compared to that, the effectiveness of vaccination was present in 92% of patients (85% fully responders, 8% partial responders) 4 weeks after the 2^nd^ dose according to our response criteria.

Vaccination against COVID-19 infection is widely recommended to all adult non-pregnant patients with IBD regardless of used therapy despite the fact that patients with immunosuppressive or biological therapy could have lower vaccine efficacy [23–25]. The efficacy of the BNT162b2 vaccine (Pfizer-BioNTech SARS-CoV-2 vaccine) used in our study was proved in a large placebo-controlled phase III trial—95% efficacy in preventing symptomatic COVID-19 infection [26]. Subsequent studies have more or less confirmed this level of protection [27–29]. The pilot study evaluated the effect of vaccination with BNT162b2 in the context of confirmed COVID-19 cases from all participants in a two-month follow-up after vaccination [26]. SARS-CoV-2 antibody levels have not been evaluated in these studies and nowadays no routine post-vaccination testing for COVID-19 is recommended and its exact role remains unclear. In our study, the degree of immunogenicity of vaccination against SARS-CoV-2 in terms of antibody production was evaluated, and not the percentage of patients who underwent COVID-19 infection after vaccination. According to our data, a clear acceleration of SARS-CoV-2 antibody levels after the 2nd dose is evident. The geometric mean levels of SARS-CoV-2 antibodies in all three groups of patients increased significantly from 34.9–40.7 before 2^nd^ dose to 500.1–676.5 4 weeks after 2^nd^ dose. The level of antibodies guaranteeing protection has not been determined by available studies; in our study, it was determined empirically according to clinical experience with this infection. This differentiation of patients is certainly not exact, but it can help us select patients with a very poor antibody response to vaccination and recommend an early alternative procedure such as revaccination with another type of vaccine. For this reason, we consider routine testing of SARs-CoV-2 antibody levels after vaccination to be rational in high-risk patients.

Responders (SARS-CoV-2 antibodies IgG 500 U/ml and more) and partial responders (SARS-CoV-2 antibodies IgG 100–499 U/ml) after 1st dose and four weeks after the 2nd dose were in our study 32,2% and 92% of patients, respectively. The main effect and necessity of the 2nd dose of vaccination for SARS-Cov-2 is clear from our data. Only a small proportion of patients in our study did not reach an antibody response after the second SARS-COV-2 dose. These data are consistent with the results of the Kennedy et al. study, which was recently carried out on patients with IBD on biological therapy [30]. Based on this study, infliximab is associated with low immunogenicity after the 1^st^ dose of the BNT162b2 and ChAdOx1 vaccines while on the other hand the 2^nd^ dose of vaccine led to seroconversion in most patients. We have also demonstrated that doses of azathioprine more than 2mg/kg or intensified regime of biological therapy are not associated with lower representation of responders and partial responders after vaccination.

A combination therapy of biological and immunosuppressive therapy could lead to attenuated immunogenicity to both vaccines in infliximab-treated patients [30]. We directly compared the differences between the three groups of patients based on their therapy (biological therapy, immunosuppressive therapy with azathioprine and combination therapy of immunosuppressive therapy with azathioprine and biological therapy) and no statistically significant differences were proved in SARS-CoV-2 IgG antibody trends. To this date, no other study has directly compared the effect of these types of therapy on immunogenicity following SARS-CoV-2 vaccination.

In our group of patients, two basic types of local reaction after vaccination were observed: local erythema and/or local pain. On the day of administration, these local reactions were observed in approximately 60% of patients after both doses. The majority of these problems was present the day after vaccination—in 75.5% of patients after the 1^st^ dose and in 65.3% of patients after the 2^nd^ dose. This trend of maximum local reactions on the day following vaccination is in correlation with another study dealing with SARS-CoV-2 vaccine adverse events [31]. According to the data of the next study, after the 1^st^ and 2^nd^ doses of BNT162b2, local reactions were present on the day of application in about 30% and 40% of patients, respectively, and in the following day in 70% and 80% of patients, respectively [27]. These results correlate with our data. In our group of patients and in terms of local reactions, the 2^nd^ dose of the vaccine was better tolerated, compared to the above-mentioned study.

A maximum of general adverse events was observed in our study on the day of administration and the following day after both doses of vaccine, in the following days there was a significant decrease of these problems. After the 1^st^ and 2^nd^ doses, a total of 41 and 42 adverse reactions were reported on day 0 and 34 and 59 adverse reactions on day 1, respectively. The most common general reactions were: fever, myalgia, arthralgia, cephalgia and fatigue. Gee et al. reported that fatigue, headache, and myalgias were present on day 0 after the 1^st^ and 2^nd^ doses in approximately 10, 10, and 5% and in 15, 10, and 5%, respectively, and on day 1 after the 1st and 2^nd^ doses in approximately 25, 50, and 15% and in 55, 45, and 50% [31]. In comparison to our data, these adverse events were also present more often after the 2^nd^ dose and with maximum on the day after vaccination, but not so often. Anaphylactic reactions following COVID-19 vaccines have been reported in 4.5 cases per million doses [31]. We reported no severe allergic reaction in terms of anaphylaxis.

Limitations of this study are:

1. Short-term study on relatively small number of patients.
2. Real effectiveness of vaccination against COVID-19 infection was not evaluated in our study. The immunogenicity of the vaccine and the production of antibodies against COVID-19 were measured.
3. The concentration of IgG against COVID-19 was measured up to the maximum limit of 1000 U / ml. This fact may affect the assessment of the median levels of IgG antibodies in our study groups in terms of underestimation of our results (not overestimation). However, it has no effect on the evaluation of COVID-19 vaccination responses.

## Conclusion

SARS-CoV-2 vaccination should be recommended for all adult non-pregnant patients with CD or UC regardless of the therapy as soon as possible. Our study proved the efficacy of vaccination against COVID-19 in patients with IBD treated by immunosuppressive and/or biological therapy. No statistically significant differences of vaccine immunogenicity were proved between these groups of patients. We also proved the safety of SARs-COV-2 vaccination, because adverse reactions following vaccination were only minor and of short duration in our study.

## Supporting information

S1 Fig Adverse events questionnaire. Questionnaire focused on the safety of the administered vaccine.
(DOCX)

S1 File Source data of this study divided into four tables. (XLSX)
