# Supplementary material for: Efficacy and safety of SARS-CoV-2 vaccination in patients with inflammatory bowel disease on immunosuppressive and biological therapy: Prospective observational study
Source: PLoS One. 2022 Sep 2;17(9):e0273612. doi: 10.1371/journal.pone.0273612 (PMC9439210; doi:10.1371/journal.pone.0273612)
Supplement: S1 Fig — Questionnaire focused on the safety of the administered vaccine. (DOCX) [file pone.0273612.s001.docx]

**A) LOCAL REACTION**

1) Pain YES – NO

Intensity of pain (1-10):

2) Redness in the injection site YES – NO

Size (diameter in cm):

**B) SYSTEMIC REACTION**

1) Body temperature in the morning: ……°C

2) Body temperature in the evening: ……°C

3) Pain of muscles YES – NO

Intensity (1-10):

4) Pain of joints YES – NO

Intensity (1-10):

5) Headache YES – NO

Intensity (1-10):

6) Severe allergic (anaphylactic) reaction YES – NO

7) Other difficulties:

8) Necessity of emergency medication:

Medicament:

Dose:

Time of administration:

**C) TOTAL EVALUATION AFTER VACCINATION BY PATIENT (0–100, 0 worst and 100 best):**

Value:
